# Putting the (R) Ural in Preceptorship

**DOI:** 10.1155/2012/528580

**Published:** 2012-05-29

**Authors:** Deirdre Jackman, Florence Myrick, Olive Yonge

**Affiliations:** Faculty of Nursing, Edmonton Clinic Health Academy, Edmonton, AB, Canada T6G 1C9

## Abstract

Rural nursing is recognized as a unique health care domain. Within that context, the preceptorship experience is purported to be an important approach to preparing safe and competent rural practitioners. Preceptorship is the one-to-one pairing of a nursing student with a professional nurse who assumes the mandate of teacher and role model in a designated clinical/contextual setting, in this case the rural setting. A research gap exists in the literature in which rural preceptorship is specifically explored. The purpose of this paper is to review preceptorship in relation to preparing nursing students specifically for the rural setting. Understanding how preceptorship as an educational model can prepare nursing students to transition to rural practice is an important endeavor. An authentic rural preceptorship may serve to influence the recruitment and retention needs for registered nurses in rural areas. A greater understanding of rural preceptorship serves to illustrate the appropriate support, socialization and contextual competence required to prepare nursing students for rural nursing practice. This paper's review may serve to highlight the research that currently exists related to rural preceptorship and where additional research can contribute to further understanding and development for authentic rural nursing preparation.

## 1. Introduction

Nursing as an applied practice requires its professional members to have a sound theoretical knowledge in addition to attaining proficiency in practice and skills within a diversity of clinical/contextual environments. To this end, nursing students during their undergraduate programs are exposed to theoretical teaching in classroom environments in unison with clinical experience, thus cementing their learning in relation to patient interactions and care. One of the established educational models, utilized to augment students' learning, is the preceptorship model which promotes the socialization, competence, and confidence of nursing students in the clinical/contextual setting as they transition from the role of the student to the role of newly graduated registered nurse [1]. 

However, the question arises that for specific environments such as the rural context, does utilization of a preceptorship model serve to provide relevant transitional learning for nursing students and what role does context play in the students' experience [2]? Therefore, the purpose of this paper is to review the literature related to “rural nursing preceptorship” and its role in preparing nursing students specifically for rural nursing practice.

## 2. Canadian Rural Context

Historically the term “rural” has been used primarily to refer to geographic location and distance [3, 4]. Research indicates, however, that the exclusive focus on the geographic interpretation fails to fully encapsulate the actual definition of the rural setting and what that term truly represents [5, 6]. Expressing rural context in simple geographic terms negates the complexity of “rural” where distance itself does not fully describe the term [7, 8]. Currently, the most common definition of rural has been divided into two categories, one is described as technical differentiation and the other is social differentiation [4]. Technical differentiation refers to geographic measurements in cartographic representation. Technical terminology can encompass statistical measurements such as those developed by Statistics Canada, vis-a-vis, Rural and Small Town (RST) which indicates populations living outside the commuting zones of larger urban centres, for example, outside Census Metropolitan Areas (CMAs) containing populations of 100,000 or more and Census Agglomerations (CAs) containing populations of 10,000 or more [9].

 In terms of health care services, location is measured in relation to travel distance to hospitals and acute or community health care centres. Distance is represented as kilometres away from urban tertiary centres [10]. However, Sorenson and De Peuter [11] indicate that the concept of “rural” has become more expansive in definition and interpretation to include social attributes such as income and education that impact the health of rural populations. Rural research, including nursing research, has expanded to encompass further understanding of rural health care and professional practice [3, 4, 12, 13]. Rural descriptors have attempted to allow social issues to be included with geographic issues to portray a more complete image of this environment thereby painting a picture of what community life is like, what the challenges of living in these communities mean, and what the needs and rights of these populations entail. In the past, rural health research has focused primarily on the accessibility of health care services, but there is a continued need to understand other health determinants and their implication on the health for these populations [9]. Research indicates that Canadian rural residents have a shorter life expectancy related to chronic conditions such as cardiovascular diseases, diabetes, and death due to motor vehicle accidents and suicide [14, 15]. However, rural communities also perceive a stronger sense of community than their urban counterparts as a contributor to their sense of well-being and sense of belonging [14]. Currently, rural communities have significant health challenges but face at times a lack of access to health care providers, most especially rural nurses, more than their urban counterparts. These percentages have not increased in sufficient amounts to bring health care provider-to-population ratios to the levels enjoyed by urban populations. There remains a shortage of rural nurses [16, 17]. Thus, it becomes important to appropriately prepare nurses for rural practice settings.

## 3. Rural Nursing Practice

Rural environments are fast paced, complex, and use an ever-increasing array of technology. These technologies include telehealth access to specialist collaboration, educational building capacities to provide interprofessional training related to emergency, cardiac, and obstetrical care [8, 18]. To that end, rural nursing practice is diverse, requiring particular foundational knowledge, practice, and skills. Rural nurses posses the specific knowledge and skills to provide expanded health care services to rural populations using a holistic approach to health care [19]. Health factors and health care services that relate to community structures, inclusive of social support, employment, education, and environmental influences are understood by rural nurses in conjunction with recognizing their fit within unique rural settings [20]. Kulig et al. [21] surveyed registered nurses practicing in Canadian rural and remote settings. They found four dominant themes. These include community characteristics, geographic location, human health and technological resources, and nursing practice characteristics. The authors suggest that these themes can contribute to a further understanding of rural settings and nursing practice.

Today, registered nurses (RNs) constitute the largest profession providing health care in a variety of rural settings [22]. Within rural communities, RNs are considered unique in their professional and personal roles [23]. There is a role blurring between the professional and personal [13]. This differs from the nursing role of their urban counterparts. Rural nurses engage in what can be described as an intertwined relationship between their personal and professional roles in the rural setting. They are community members who are living and practicing amongst their interprofessional colleagues, family, and neighbours in a closer relational proximity than occurs in the urban setting.

 Nurses embody their rural practice [21]. This dual role affords nurses the opportunity to immerse themselves in the context of their practice. Kulig [5] describes this integration of the professional and the personal role as central to creating an authentic knowledge of rural practice. Rural nurses who practice within the various rural settings are patently aware of the unique needs and health care requirements. While there are challenges in rural practice, rural nurses and community members also highlight the positives of living within a rural community such as feeling a sense of open space and a feeling of connection to values, beliefs, and sense of community [24].

Historically and currently, it is rural nurses who have been providing essential care to rural populations [25–27]. Rural nurses, together with their community members, can identify specific health care and other requirements, including how these needs should be met [13], thus creating political and personal advocacy for the unique needs of rural communities. RNs have been and continue to be a linchpin in rural health care. However, recruitment and retention in rural settings remains a challenge [16, 17]. With the ongoing, critical need for RNs, demographic data indicate that the global and Canadian age of RNs is of continuing concern. Many RNs are at or near retirement [17]. Currently and in the future, this shortage implies a decreasing number of experienced RNs to fill the required vacancies. In the rural areas, there is an even greater urgency to recruit and retain RNs. In Canada, it is noted that rural RNs are older than their urban counterparts, the population ratio of patient to nurse is greater, and the shortage of these health care professionals is more acute in the rural communities who are left to cope with diminished access to health care professionals, most especially RNs [15, 16]. Rural communities face an array of complex health care needs, and with the increasing reduction of tertiary care beds available in the urban hospitals, rural patients health needs, including increased complexity of care, are provided by rural nurses and other health care professionals [17]. Rural communities indicate that they deserve equal access to health care needs including the availability of well-prepared rural RNs.

Education is key in order to prepare RNs to practice in the rural settings and should be considered for existing and future recruitment and retention strategies [28]. The specific needs of the rural settings need to be understood, and educational requirements including preceptorship need to be formulated and augmented to address rural nursing preparation to ensure both short- and long-term rural health care delivery. Thus, it becomes imperative to recognize the need to educate undergraduate students who are exposed to rural clinical environments within their nursing programs to ensure adequate preparation for practice in the rural setting [29]. Rural preceptorship may provide such an educational model to promote authentic learning. Rural nurses possess the knowledge and practice requirements necessary in the rural settings. Their experience and expertise can therefore provide relevant teaching and role modeling in preparing nursing students for all aspects of the rural setting, through the preceptorship process. The literature findings indicate that nurses who feel prepared to practice in the rural setting will be more likely to stay and practice over the long term [10, 30].

## 4. Rural Preceptorship

Some of the underlying principles of preceptorship date back to the teachings and writings of Florence Nightingale [1]. Nightingale posited that it was necessary for experienced nurses to teach students how to provide nursing care, through guidance and facilitation of learning. Myrick [31] indicates that nurse educators recognize that as a professional discipline nursing knowledge is embodied in its application to practice. Clinical experience is the cornerstone of socialization of novice nurses into the profession [32, 33]. The preceptorship model has emerged as central to this teaching/learning approach [34]. Preceptorship models purport to provide formal learning for the students by pairing them with an expert RN with experience in the clinical/contextual setting. The key role of the preceptor is that of teacher and role model. These knowing, showing, and doing are what nurture the nursing student in the clinical context. O'Malley et al. [35] suggest that nurturing the student to become a competent and confident practitioner in a specific setting, in this case the rural setting, allows a strong sense of connection to the environment and illuminates the required expectations of the nursing care for the particular populations being served. It is the preceptor who possesses the clinical experience, knowledge, and skills to be able to provide strong role modeling and teaching [36, 37]. Support of the nursing student's learning can allow the student to experience the benefits of preceptorship. The student who experiences a positive preceptorship is supported to facilitate successful entry to practice [38, 39]. According to Manahan and Lavioe [10], a student entering a practice environment competently and safely creates a feeling of belonging to the profession and of being a capable practitioner. This sense of belonging can contribute to recruitment and retention where a practitioner wants to apply for employment and stay over the long term. Thus, preceptorship as a model of education has been supported by many scholars [40–42].

A review of the literature confirms the well-examined role of preceptorship and its influence on learning and socialization of the novice nurse [1, 23, 31, 38, 43, 44] (Hegney, McCarthy, Rogers-Clark, and Gormann, 2002). However, a lack of research is available regarding rural preceptorship. Many studies indicate the benefits of preceptorship to educate students in the clinical setting but do not articulate or include the substantive area of rural context [1, 31, 34, 36, 45–47]. Currently, the small number of existing studies which have been conducted and related to rural preceptorship suggests particular benefits for clinical preparation of students, including nursing students, in the rural context [2, 10, 19, 23, 28, 48–50]. These studies serve to answer some of the questions related to rural preceptorship and stimulate researchers to ask additional and unique questions. The findings of these studies indicate the unique practice of rural nursing, the unique needs of rural populations, and the need to incorporate specific findings such as culture, professional boundaries, conflict management, and undergraduate and graduate education as important elements of current and future rural nursing education. Existing research related to rural preceptorship highlights the influence of preceptorship to accomplish teaching/learning components necessary for rural nursing practice. Preceptorship is an important key to preparing undergraduate nursing students who are placed in rural settings [51]. Preceptorship in this setting draws on the rural clinical/contextual expertise of the nurse preceptor to role model, teach, supervise, and evaluate to ensure preparation of the preceptee (nursing student) for the specific rural context. Altmann [36] and Letizia and Jennrich [47] note that the preceptor should be selected on criterion including clinical/contextual expertise in rural nursing practice and an ability to communicate well to provide teaching, leadership, skills, and role modeling necessary in this complex and unique practice setting.

Yonge [28] asserts that it is important to offer a relevant vehicle to support the educational and practical preparation of the rural nurse as s(he) enters the rural environment. Yonge contends it is important to examine the rural setting as it relates to and influences the experience. The rural context/setting which differs from other practice/clinical settings provides an additional component to preceptorship [52]. The author suggests that more research is required related to preceptorship and the rural setting to close the research gap and to inform rural educational preparation in undergraduate nursing programs. Yonge further suggests that rural preceptorship can combine the tenets of preceptorship with the tenets of rural nursing to elucidate an experience that speaks to both practice preparation and context. In the case of rural preceptorship, the prefix of rural is a necessary antecedent of preceptorship. Rural preceptorship needs to reflect a direct rural focus [53]. Examination of the literature indicates that rural preceptorship differs from urban preceptorship. For example, Sedgwick and Yonge [49] note that the culture in rural hospital settings has a strong community environment that presents itself in a team approach to nursing practice that differs from urban hospital settings. Sedgwick and Yonge further state that this particular culture of team nursing influences the dynamics of rural preceptorship. Rural nurse(s), other than the preceptor, are present in the rural setting during a nursing student's preceptorship. Although their relationship is not a formal one such as exists between the members of the preceptorship triad, they will have interactions with the nursing student throughout the preceptorship experience. Rural setting creates a team or community approach to rural preceptorship that includes significant interactions with other nurses in addition to the formal relationship between the preceptor, student, and faculty member [49].

To date, researchers have conducted a number of studies examining the multiple facets and definitions of “rural” [4, 7, 8, 12, 23]. This rural research serves to facilitate understanding of the rural setting and the nursing practice required [12, 54]. Rural-oriented researchers indicate the continued need to provide well-prepared nurses for rural practice settings but do not articulate or address how to provide this preparatory education. A gap therefore exists in the literature specifically related to undergraduate nursing education for rural practice. Research related to particular aspects of rural preceptorship is necessary to ascertain how the role and influence of the rural nurse preceptor contributes to the support and preparation of nursing students for practice in the rural setting.

Research related to rural nursing indicates that it is not designated a “speciality practice.” Furthermore, specific educational components related to rural practice are absent [55, 56]. Educational preparation becomes a transplantation of urban programs without inclusion of the unique needs for rural nursing practice preparation. Crooks [23] suggests that rural nursing education and practice preparation requires specific knowledge and practice skills that are ignored and remain unacknowledged. She calls for recognition of rural nursing practice as a unique domain of practice that requires thoughtful and relevant educational preparation. It is important not to negate the rural context, but to recognize it as central to informing the practice of rural nurses. As in the past, rural nursing practice is considered to be expansive in terms of the breadth and depth of knowledge and the skills required to provide the necessary nursing care [12]. Because of the contextual influence on rural nursing practice, the ability to role model and teach nursing students must be provided by practicing rural nurses. The influence of the rural context and the experience of rural nurses are thus central in the preceptorship process in contributing to the legitimate and specific preparation of nursing students entering the rural setting.

 Kenny and Duckett [19] highlight the importance of educational preparation for rural nurses. The authors suggest it is this specific education for practice that supports the retention of rural nurses who are competent and confident. Because of the connection of rural nurses to their professional practice and rural communities, their ability to articulate and role model rural nursing to nursing students is strong and considered essential to rural preceptorship. It is the rural nurses who provide the expert practitioner role in the preceptorship relationship for students who are seeking to practice in the rural setting.

Many students who have an interest in rural nursing and would like to practice in a rural community have been educated in urban nursing programs, using urban clinical settings. Their curriculum does not address or include any aspects of what may be unique to rural nursing [2, 57]. Therefore, they might then be expected to practice with competence upon graduation in the rural context, but their exposure and education are from an urban undergraduate nursing program where rural experience and educational support are absent. According to Charnley [58], it is only after graduation that these novice practitioners become immersed in rural context and have to begin to learn to navigate a complex and daunting practice setting. Their learning is obtained while they are practicing. This vicarious exposure, experience, and support for novice rural nurses create an element of anxiety and stress. Concomitant, they are required to become competent and confident as they practice [11]. Thomlinson et al. [24] indicate that this lack of preparation within undergraduate nursing programs leads many to feeling overwhelmed, choosing to move away from the rural areas or out of nursing. In order to allow nurses who wish to practice in rural areas to feel educationally supported for the rural context, preceptorship can offer a sound vehicle to accomplish preparation for clinical, contextual, socialization, and competent practice.

## 5. Conclusion

Sedgwick and Yonge [2] suggest the importance of studies to explore how preceptorship can contribute to the preparation of nurses specifically for the rural setting. The authors conclude that understanding the cultural climate that occurs in rural preceptorship is important but note that further examination of rural preceptorship is also required. Yonge [28] posits that such research can inform rural nursing preparation and contribute to shaping the experiences of those involved. Rural preceptorship can provide a vehicle for the socialization of nursing students to the rural setting [1, 59].

Since the 1980s, preceptorship has been successfully used by nursing programs to educate and prepare students to transition into practice [31]. A cornerstone of the preceptorship model is the emphasis on clinical/contextual preparation. It is the ability of the experienced preceptor and the experienced faculty member to provide guidance for the student to be successful. The literature on the benefits of preceptorship to allow a student to feel competent, confident, and socialized as s(he) enters the practice areas is well documented [35, 59–61]. However, the unique aspects of rural preceptorship are not well documented. There is a void related to how the role of rurality impacts the preceptorship experience and a gap exists in the literature specific to rural preceptorship. The literature related to nursing and “rural preceptorship” and more specifically to “rural nursing preceptorship” in preparing undergraduate nursing students is limited. Rural preceptorship studies have focused on certain aspects of preceptorship in the rural setting including the roles of the preceptor and the student [40, 47, 62] (Yonge, Krahn, Trojan, and Reid, 2002). Researchers have examined issues such as conflict in the preceptorship relationship [63]. Yonge et al. [63] examined preceptors and students' perceptions of the rural preceptorship experience in relation to factors that can cause challenges for the preceptor and preceptee such as geographic distance, maintaining communication with faculty members, integrating students into rural practice, weather conditions, and a lack of resources. Leipert et al. [64] concurred that weather conditions, direct communication, and access to care in rural communities pose unique challenges within rural preceptorships. Sedgwick and Yonge [2] have examined the cultural climate of the rural context and its influence on the preceptorship experience. While these studies examine aspects of rural preceptorship, additional research is needed.

Rural preceptorship can provide the necessary teaching/learning processes that combine theoretical learning and contextual/clinical learning with the required focus on rural nursing practice. Because rural nursing is unique, it is essential to prepare rural nurses educationally and formally with a rural as opposed to an urban focus. An authentic rural preceptorship can foster rural socialization and the critical thinking germane to such a context. In order to achieve this authenticity within preceptorship, it is necessary to select preceptors who not only possess communication attributes but also commitment, experience, and leadership within rural nursing [31] (Kenny and Duckett, 2005).

The authors of this review paper note that there remains a void in the literature of what constitutes a rural preceptorship. It is incumbent upon nurse researchers to conduct additional studies which examine the rural preceptorship process, thus creating evidence to support and develop educational content relevant to the preceptorship model and the rural context. These attributes will ensure the merging of the rural aspects of nursing within preceptorship. If positive and pertinent rural preceptorships can be achieved to support practicing in a rural environment, the ability to provide rural nurses, who will stay because they feel connected and safe to practice, can be a reality.
